# Medico-legal management of healthcare-associated infections: a cost-effectiveness analysis in an Italian tertiary hospital

**DOI:** 10.3389/fmed.2024.1430625

**Published:** 2024-08-27

**Authors:** Simone Grassi, Maddalena Grazzini, Marta Guerini, Giorgio Bertana, Linda Pompeo, Diana Paolini, Fabrizio Niccolini, Martina Focardi, Vilma Pinchi

**Affiliations:** ^1^Department of Health Sciences, Forensic Medical Sciences, University of Florence, Florence, Italy; ^2^Hospital Infection Prevention and Control Unit, Florence Careggi University Hospital, Florence, Italy

**Keywords:** healthcare-associated infections (HAI), medical malpractice claims, medical malpractice, legal medicine, compensations

## Abstract

**Introduction:**

Healthcare-associated infections are the main reported adverse event in healthcare, with significant economic costs that include those caused by medical malpractice claims. In Italy, there is a fault-based compensation system, but in this specific field, the burden of proof on the hospitals is particularly heavy. Hence, we aimed to verify the economic impact of the inclusion of experts in hospital infection surveillance into internal committees for claims assessment and to evaluate what would have been the economic impact of a mandatory no-fault system rather than the current system.

**Materials and methods:**

We compared two 4-year periods (T1: 2015–2018 and T2: 2019–2022), investigating the medical malpractice claims related to healthcare-associated infections in a large tertiary public hospital in Florence, Italy. Decisions of the internal committee, evolutions of the claims after the decision, and conclusions of the claims were registered. No-fault system simulations were used to evaluate the cost-effectiveness of the model.

**Results:**

We observed a decrease in the number of claims after the implementation of infection prevention and control (IPC) experts into the committee (a 24% decrease in rejections and a 19% increase in admissions). We found a 6806.98 euros difference (not statistically significant) in compensations in T1 and T2. Moreover, our simulations found that a no-fault compensation system – if alternative to the traditional fault-based approach – could lead to gains or losses for the plaintiffs depending on the approach chosen. (We observed a 52% mean decrease in compensations with a 150000 euros maximal indemnity and a 134% mean increase with an indemnity tailored considering also life expectancy).

**Discussion:**

Introducing experts in IPC into hospital committees for medico-legal claims management has proven to be cost-effective, offering a no-fault compensation system as an alternative to the traditional fault-based approach, supported by a properly evaluated maximal indemnity. Due to the limitations of our models, multicentric studies are recommended to verify our results.

## 1 Introduction

Healthcare-associated infections (HAIs) are the main reported adverse event in healthcare, with a prevalence in high-income countries ranging from 3.5 to 12% (1). They have a very significant economic impact, increasing in-hospital mortality and length of stays, direct costs of care, and, in the European Economic Area, causing annually 501 disability-adjusted life years per 100000 habitants (2–4). Most HAIs are of (at least potential) medico-legal interest since they are often preventable through multimodal strategies enforced at organizational, structural, and individual levels (5). A milestone of an infection prevention and control program (IPC), according to the World Health Organization, is HAI surveillance (6). In particular, in the European Union, the supranational HAI surveillance network (HAI-NET) performs a point prevalence survey of HAIs in acute care hospitals every 5 years, and HAI surveillance programs are implemented at national, regional, and local levels (1).

When costs related to HAI are considered, the economic burden due to HAI cases of alleged medical malpractice should be taken into account.

A recent ruling of the Italian Supreme Court (6386/2023) compels hospitals to compensate patients who suffered from HAI unless they can prove 13 items (Table 1).

**Table 1:** Hospital burden of proof (according to the Italian Supreme Court).

Compliance with protocols of the following: Disinfection and sterilization of materials and the hospital environment; Laundry management; Waste management; Distribution of food and water; Preparation, storage, and use of disinfectants; Monitoring of air quality; Control and limitation of visitors; Occupational injury control strategies and vaccination; HAI surveillance and infection disease reporting; Use of microbiological data for HAI surveillance; Alert organisms surveillance. The ratio between hospital staff and inpatients/outpatients The time when each of the aforementioned risk management activities was performed

This significant burden of proof requires to structure a complex and articulated line of defense, a challenge for the Italian system in which, when the hospital has no insurance coverage or has high-deductible insurance coverage, medical malpractice claims are directly managed by committees composed of experts in tort law and legal medicine (7, 8). In Italy, there is a restorative justice system (no punitive damages are allowed), and permanent impairment is generally the main kind of non-economic damage related to medical malpractice. It is evaluated by an expert in legal medicine who, considering the functional history and the medico-legal physical examination of the patient, uses numerical coefficients to rate the permanent impairment of global individual functioning (the full psycho-physical integrity is equal to 100%) (9). Italian Law 210/1992 regulates no-fault compensations in cases of impairments caused by mandatory vaccinations or severe HAI (such as HIV) contracted through blood transfusions, but the patient who asks for these indemnities still has the right to also legally ask for fair compensations based on proof of fault.

This study aimed to investigate the economic impact of medical malpractice claims for HAI in a tertiary public hospital and the impact of an in-house strategy to manage this issue. In detail, the primary endpoint of this study is to verify whether the inclusion of experts in IPC, specialized physicians from the Hospital Infection Prevention and Control Unit, into hospital committees for claims assessment has an impact on their performances and the mean cost of claims. The secondary endpoint is to evaluate what would have been the economic impact of hypothetical mandatory no-fault systems on public health system finances and plaintiffs rather than the fault-based system currently valid in Italy.

## 2 Materials and methods

We considered the cases in which a patient or his/her heirs requested compensation for an HAI to the Careggi University Hospital (a public 1200-bed tertiary hospital in Florence, Italy). The data source was the database of the Evaluation Committee of medical malpractice claims (MEC), an internal committee composed of hospital experts in legal medicine and tort law (loss adjusters and lawyers), whose mission is to determine whether and how much compensation should be awarded to claimant patients. In case of alleged damage due to a HAI, most compensations are related to patients’ permanent impairment or death caused by the infection. Experts in IPC have constitutionally participated in MEC meetings since 2019, participating in the analysis of the cases and producing epidemiological reports (ER) for medico-legal purposes in cases of alleged HAI when the MEC evaluated that mere technical argumentations were not sufficient for proper medico-legal defense.

For each case of HAI-related alleged medical malpractice claim (MMC), this information was collected from the database of the MEC: the decision of the MEC (admission or rejection of the claims), the evolution of the MMC after the MEC decision (desistance of the plaintiff or civil proceeding), and the conclusion of the MMC (also in economic terms). If the plaintiff withdrew the request before the MEC decision, this information was still noted. Although data were available since 2010 (when the hospital decided to introduce the MEC), we focused on two-time intervals composed of the same number of years (T1: 2015–2018; T2: 2019–2022) because, in 2019, the MEC started using ER for MMC evaluation and hospital legal defense. In period T2, three cases of SARS-Cov-2 infection were included.

In detail, the investigated categorical variables in T1 and T2 were as follows:

1. Decisions made by the MEC: Admissions (A) and rejections (R). D indicates when the plaintiff desisted before the MEC decision;
2. Progression of claims after the MEC decision: Cases closed by MEC (C) and cases progressed into civil proceedings (P);
3. Concordance between the MEC decision and the civil court decision: concordant (Co) and discordant (Di) decisions.

Finally, the economic compensations (set in an extrajudicial or judicial context) were noted, together with the percentage of permanent impairment caused by HAI.

STATA software (v. 18.0, StataCorp LLC, US) was used to perform a t-test to compare mean compensations in T1 and T2, with the cut-off of statistical significance set at *p* = 0.05.

Finally, we made three simulations (whose limitations are reported in the discussion) in order to predict what would have been the increase or decrease in the expenditure of the public system for MMC and the mean “loss” or “gain” for plaintiffs in three different scenarios:

1. In this scenario, it is hypothesized that the same amount of money actually paid by the public system is distributed using, as the sole distribution criterion, the decimal coefficient of permanent impairment evaluated by the expert in legal medicine. In this model, the maximal indemnity, i.e., the sum of money that could have been paid to a single patient with a 100% permanent impairment (in order to simplify the inferential model, death was also equaled to 100% impairment), is calculated considering the amount of money actually paid by the public system for all the cases of HAI divided by the sum of the coefficients of permanent impairment.
2. In this scenario, the maximal indemnity (defined as in 1) is set at 150000 euros (the indemnity given to the close relatives of fatal cases of vaccinations following Law 210/1992), and the indemnity paid to each patient is obtained by multiplying the maximal indemnity by the coefficient of permanent impairment of the specific case.
3. In this scenario, the maximal indemnity (same definition as before) is obtained by multiplying the Italian mean per-capita Gross Domestic Product (28200 euros) by the life expectancy when the medical malpractice was claimed. The individual indemnity – as before – is calculated by multiplying the maximal indemnity by the permanent impairment coefficient. The Italian mean per-capita Gross Domestic Product and life expectancies were obtained from the Italian National Institute of Statistics.^1^

## 3 Results

Regarding the primary endpoint, in T1 and T2, the hospital received 46 and 35 HAI-related MMC, respectively. (Note that hospital medical malpractice claims in general were 669 in T1 and 522 in T2.)

**Figure 1:**
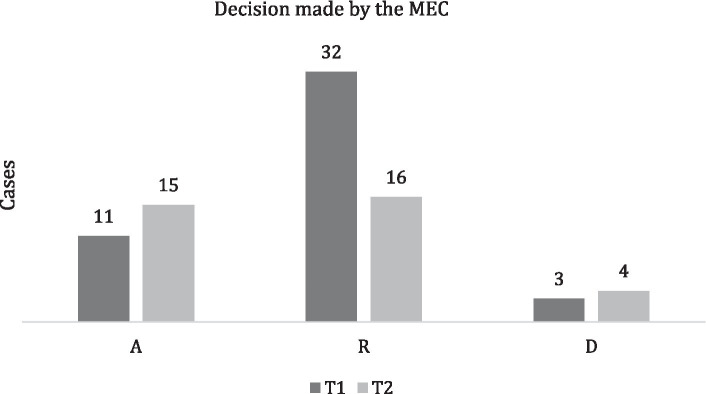
Decision made by the MEC. Admissions (A), rejections (R), and plaintiff desisted before MEC decision (D).

In T1, there were 32 rejections (70%) and 11 admissions (24%) made by the MEC and 3 cases of pre-decision desistance (6%), while in T2, there were 16 rejections (46%), 15 admissions (43%), and 4 (11%) cases of pre-decision desistance (Figure 1).

In T1, MEC managed to directly close (accepting or rejecting the claim) 17 cases (37%) and, as said, 3 cases (6%) were closed because of desistance, while in 26 cases (57%), the failure to agree on compensation led to a civil proceeding. A total of 10 cases rejected by the MEC did not progress.

**Figure 2:**
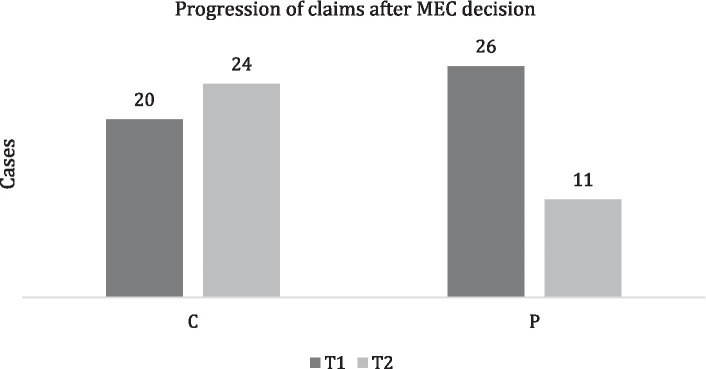
Progression of claims after the MEC decision. Cases closed by MEC (C) and cases progressed into civil proceedings (P).

In T2, MEC managed to directly close 20 cases (57%); while in 11 cases (32%), there was a civil proceeding. However, 11 cases rejected by the MEC did not progress (Figure 2).

**Figure 3:**
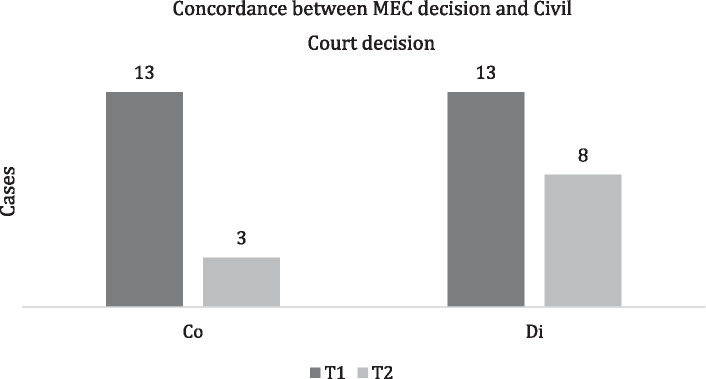
Concordance between the MEC decision and the civil court decision. Concordant (Co) and discordant decisions (Di).

In total, 26 MMC in T1 and 11 MMC in T2 led to civil proceedings, and concordance with MEC decisions was found in 13 cases (50%) and 3 cases (27%). In T2, in all eight cases in which the proceeding outcome was different from the MEC decision, an ER was not formally attached (Figure 3).

**Figure 4:**
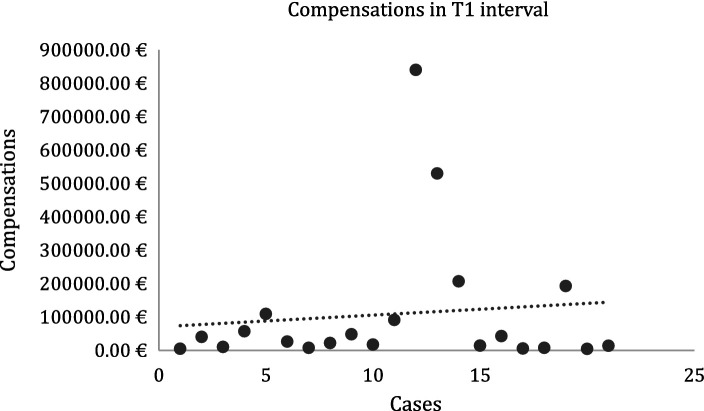
Compensations in T1 interval.

**Figure 5:**
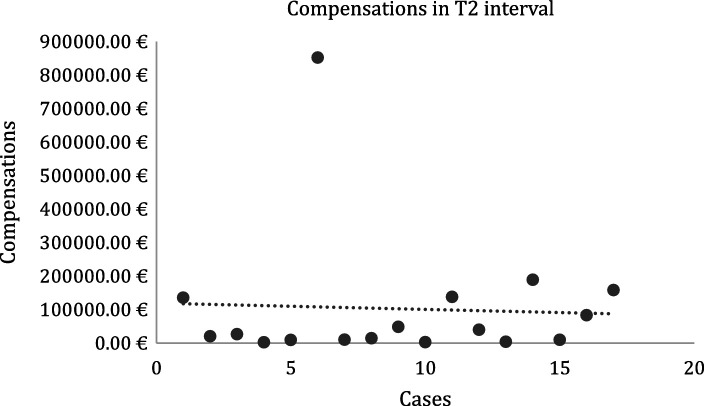
Compensations in T2 interval.

Regarding mean compensation comparisons, the T1 and T2 datasets were unpaired and presented unequal variances. The mean value for T1 was 109018.78 euros (Figure 4) and for T2 was 102211.80 euros (Figure 5) (difference between T1 and T2 values: 6806.98 euros). A two-sample t-test found a *p*-value of 0.92 (> 0.05).

The secondary endpoint was evaluated through limited simulated scenarios:

- In scenario 1, we observed a 74% mean increase in compensations (maximal indemnity: 540514.78 euros).
- In scenario 2, we observed a 52% mean decrease in compensations and a 72% decrease in public expenditure (1053000 euros vs. 3794413.77 euros; maximal indemnity: 150000 euros).
- In scenario 3, we observed a 134% mean increase in compensations and a 67% increase in public expenditure (6346790.88 euros vs. 3794413.77 euros; mean maximal indemnity: 835949.23 euros).

## 4 Discussion

To the best of our knowledge, this is the first study to report a cost-effectiveness analysis of a multidisciplinary approach to HAI-related MMC.

As reported by Norelli et al., MECs can transform their organizational structure in order to enhance their economic performance (7).

In our study, comparing T1 (2015–2018) and T2 (2019–2022), we observed a decrease in the number of MMC. The fact that Italian Law grants a significant time (even higher than 10 years) to present an MMC to a health institution may limit the strength of this evidence, but in Italy, the main time interval between the event and the MMC has been reported to be 1.69 years (10). However, our institution completely retains the medico-legal risk (i.e., chose not to have insurance coverage for MMC), and the observed trend is concordant with those reported by other national institutions that retain most of the risk, as with the trend of our institution in the last decades (7, 8, 11). On the other side, Bonetti et al., who studied data from Italian insurance brokers, observed a linear increase in MMC (including those unrelated to HAI) over time, particularly pronounced in Tuscany (the region where our hospital is) (10).

We also found that the implementation of IPC experts into the team of hospital claims management experts was associated with a change in decision-making performed by MEC (a 24% decrease in rejections and a 19% increase in admissions). These data can be interpreted in different ways, but the likeliest explanation is that direct analysis of IPC data may be an objective indicator of whether the hospital can be considered liable for failing to prevent HAI, and then hospital decision-makers can be more confident to opt for early dispute resolution rather than to take chances in an expensive civil proceeding. The composition of the MEC is a crucial determinant of its performance; as shown by our data, the inclusion of experts in the IPC has had a sound impact on decision-making. These experts may be of particular help also in sub-classes of HAI of particular medico-legal complexity, such as SARS-CoV-2 infections. Indeed, as previously underlined, these conditions often imply specific medico-legal issues (12, 13). In our cases, only three cases concerned SARS-CoV-2 infections, and in all the cases, the MEC, also considering the ER, opted for rejection, with desistance in two cases and successful defense in a civil proceeding in the last case. Finally, implementing MEC – as in our institution – with a clinical risk manager is likely to produce a beneficial system of incident reporting that allows to promptly intercept and address organizational and individual failures (8).

T2 was also associated with a 25% increase in cases closed by MEC, an improvement in performance that can be at least partly explained by the increase in the number of accepted requests, despite the evaluated implementation could have discouraged the plaintiff from progressing through, for instance, the production of ER. However, the studied intervention is also associated with a 23% decrease in concordance between MEC and civil court decisions, meaning that when a case is not closed by the MEC, it is more likely to lead to compensation for the plaintiff. In all cases in which the proceeding outcome was different from the MEC decision, an ER was not formally attached. This evidence could induce one to think that the absence of formal reports could jeopardize the strength of the hospital’s line of defense. Regarding the likelihood of civil proceedings in these cases, in general terms, Hwang et al. reported that infectious diseases, despite being the main cause of MMC, were associated with the lowest success rate of litigations (14). However, as reported by Sage et al., litigations were strongly associated with the success of the plaintiff (15). Therefore, looking at our experience, increasing the performance of MEC and containing costs should be the two main goals of the hospital in order to avoid at-risk litigations. In this regard, it is interesting to observe the slight increase in plaintiffs’ desistance before and after MEC decisions.

Regarding economic aspects, we failed to find a statistically significant difference in compensations due in T1 and T2, despite a 6806.98 euro difference between the mean values reported. This result could be read as an indicator that the studied intervention radically changed the hospital’s decision-making progress without an economic benefit. However, a decrease in average compensation is *per se* an indicator of good hospital performance if it is considered that in the US, China, and Italy, an increase in average payouts for MMC has been reported in the last 30 years, particularly in the last decade (8, 10, 11, 16, 17). Moreover, focusing on HAI-related MMC, it should be noted that they often represent most of the MMC costs in tertiary hospitals (11).

Regarding the secondary endpoint, we made three simulations. These simulations were intended to envision whether no-fault compensations (as a mandatory alternative to the current system) would benefit plaintiffs and the public system. These simulations were highly limited, mainly because 1) in a no-fault system, it is likely that far more patients would ask for economic indemnities (since compensation would not depend on the proof of actual failures of the hospital), 2) compensations in Italy are calculated considering many other factors (e.g., temporary impairment and medical and legal expenses), and 3) the economic value of the coefficient of personal impairment is not homogeneous neither in absolute nor in relative terms (i.e., it can be personalized considering exceptional characteristics of the case, tends to be higher when the plaintiff is younger, and its increase with the increase in persona impairment is not proportional). That being said, in our study sample, adopting a compensation method based on relatively low caps-on-damage was associated with a foreseeable relevant decrease in compensation but also with a more significant decrease in public expenditure. In general terms, the trade-off between individual and public system economic interests (and, in general, between microeconomic and macroeconomic factors) is complex since different maximal indemnities relate to significantly diverse outcomes. Hence, a no-fault system could reduce legal expenses and the time required to receive compensation, but, as shown, it has several limitations. A possible solution could be obtained by capping non-economic damages, as proposed by some authors for medical malpractice (18).

Finally, regarding the use of HAI surveillance information to support decision-making in MMC management, some ethical and legal issues should be discussed. A cornerstone of good HAI surveillance is efficient incident reporting systems, and, in this regard, the Council of the European Union recommends (Council Recommendation of 9 June 2009) that they must be blame-free. However, using this information for medico-legal purposes enables hospital decision-makers to identify profiles of gross negligence. Proof of gross negligence can be used by public authorities or private institutions (e.g., insurance companies) to “penalize” the involved practitioners, for instance, by significantly increasing the insurance premiums or, in countries like Italy, by compelling the physician to compensate the institution for (at least part of) its loss. This trade-off between the interests of health institutions and physicians must still be evaluated in light of the management and economic benefits. Moreover, according to current evidence, paid MMC is not a random event, and thus the use of HAI surveillance information is also fundamental for the hospital to identify misconducts or organizational failures that can be promptly addressed through targeted interventions (19, 20).

## 5 Limitations

Our study has several limitations. The monocentric study design limited the volume of data, thus future multicentric studies are recommended. Moreover, the unpaired sets of categorical variables with unequal sample sizes impeded to reliably perform parametric statistical testing to verify whether the variations are statistically significant. Meanwhile, the sets of continuous variables considered for the t-test had different sizes. In general, the main limitation was the small sample size, a limitation due to the fact that, up to date, no Italian institution has reported the use of ER for medico-legal purposes in scientific literature. Increasing sample sizes (for instance, by designing multicentric studies) should then be suggested.

## Data availability statement

The original contributions presented in the study are included in the article, further inquiries can be directed to the corresponding author.

## Author contributions

SG: Writing – review & editing, Writing – original draft. MGr: Writing – review & editing, Writing – original draft. MGu: Writing – review & editing, Writing – original draft. GB: Writing – review & editing, Writing – original draft. LP: Writing – review & editing, Writing – original draft. DP: Writing – review & editing, Writing – original draft. FN: Writing – review & editing, Writing – original draft. MF: Writing – review & editing, Writing – original draft. VP: Writing – review & editing, Writing – original draft.

## Footnotes

^1^ www.istat.it/en/
